# H_2_ production pathways in nutrient-replete mixotrophic Chlamydomonas cultures under low light. Response to the commentary article “On the pathways feeding the H_2_ production process in nutrient-replete, hypoxic conditions,” by Alberto Scoma and Szilvia Z. Tóth

**DOI:** 10.1186/s13068-017-0801-5

**Published:** 2017-05-05

**Authors:** David González-Ballester, Jose Luis Jurado-Oller, Aurora Galván, Emilio Fernández, Alexandra Dubini

**Affiliations:** Departamento de Bioquímica y Biología Molecular, Facultad de Ciencias, Universidad de Córdoba, Campus de Rabanales, Edif. Severo Ochoa, 14071 Córdoba, Spain

**Keywords:** Chlamydomonas, Hydrogen, DCMU, Acetate, Algae, Biofuels, Biomass, Low light, Oxygen

## Abstract

**Background:**

A recent Commentary article entitled “On the pathways feeding the H_2_ production process in nutrient-replete, hypoxic conditions” by Dr. Scoma and Dr. Tóth, Biotechnology for Biofuels (2017), opened a very interesting debate about the H_2_ production photosynthetic-linked pathways occurring in Chlamydomonas cultures grown in acetate-containing media and incubated under hypoxia/anoxia conditions. This Commentary article mainly focused on the results of our previous article “Low oxygen levels contribute to improve photohydrogen production in mixotrophic non-stressed Chlamydomonas cultures,” by Jurado-Oller et al., Biotechnology for Biofuels (7, 2015; 8:149).

**Main body:**

Here, we review some previous knowledge about the H_2_ production pathways linked to photosynthesis in Chlamydomonas, especially focusing on the role of the PSII-dependent and -independent pathways in acetate-containing nutrient-replete cultures. The potential contributions of these pathways to H_2_ production under anoxia/hypoxia are discussed.

**Conclusion:**

Despite the fact that the PSII inhibitor DCMU is broadly used to discern between the two different photosynthetic pathways operating under H_2_ production conditions, its use may lead to distinctive conclusions depending on the growth conditions. The different potential sources of reductive power needed for the PSII-independent H_2_ production in mixotrophic nutrient-replete cultures are a matter of debate and conclusive evidences are still missing.

## Background

The alga *Chlamydomonas reinhardtii* (Chlamydomonas throughout) is able to perform H_2_ photoproduction, as well as fermentative H_2_ production. Two distinct pathways have been described to explain H_2_ photoproduction. One of them, termed as direct pathway or PSII-dependent pathway, requires the participation of the entire photosynthetic electron chain, including PSII and PSI. Electrons originated from the water photolysis at the level of the PSII reach the PSI where they are transferred to the ferredoxin 1 (FDX1) and are finally donated to the primary hydrogenase of Chlamydomonas, HYDA1. The necessary participation of the PSII in this pathway implies that O_2_ is produced simultaneously with H_2_. Since hydrogenases are very sensitive to O_2_, under regular growth conditions, this process is very transitory, and H_2_ production quickly ceases as O_2_ accumulates. This pathway might have an important physiological role during the dark to light transitions of the cells. An alternative pathway termed as indirect pathway or PSII-independent pathway can also lead to H_2_ photoproduction. In this case, electrons do not originate from water photolysis at the PSII level but from a non-photosynthetic reduction of the plastoquinone (PQ) pool. It has been shown that a plastoquinone-reducing type II NAD(P)H dehydrogenase (NDA2) plays a crucial role in this process. In this pathway, electrons flow from the PQ pool to the PSI, and similarly to the PSII-dependent pathway; they are finally donated to the HYDA1 via FDX1. Note that this pathway does not require the participation of the PSII, and no O_2_ is produced. Finally, Chlamydomonas is also able to produce H_2_ linked to fermentative pathways. In this case, electrons are donated to HYDA1 from the activity of a Pyruvate Ferredoxin Reductase (PFR), which catalyzes the oxidation of pyruvate to acetyl-CoA under anoxic conditions. PFR activity is coupled to FDX1, which accepts the electrons from the catalyzed reaction and donates them to HYDA1. It has been shown that PFR is also able to use, in addition to pyruvate, oxaloacetate as a substrate (reviewed in [1]).

Chlamydomonas is able to uptake acetate and uses it as a carbon source. Mixotrophic and autotrophic growth conditions refer to the presence or absence of acetate, respectively. Importantly, most studies about H_2_ production in Chlamydomonas have been done using Tris-Acetate-Phosphate (TAP) medium, and it is well known that acetate strongly enhances H_2_ production in Chlamydomonas (reviewed in [1]).

The present Commentary Article is a response to the Commentary Article written by Scoma and Toth (Biotechnology for Biofuels, 2017). Here, we will discuss the few available data about the photosynthetic H_2_ production in mixotrophic nutrient-replete cultures incubated under low light conditions [2, 3], particularly the role of the PSII-dependent and -independent pathways. Moreover, we will discuss and try to clarify the available literature concerning the contribution of the PSII-independent pathway to H_2_ production, in both mixotrophic nutrient-deplete and -replete medium, when using the PSII inhibitor DCMU.

## Main text

### Comparison of the recent data obtained for H_2_ production in TAP cultures incubated under low light: commentaries on the publications of Scoma et al. [2], Jurado-Oller et al. [3], and responses to the Commentary Article of Scoma and Toth (2017)

The commentary article published by Scoma and Toth (Biotechnology for Biofuels 2017) discussed the pathways feeding the H_2_ production process in nutrient-replete Chlamydomonas cultures incubated under low light, and highlighted the different results and data interpretation obtained by Scoma et al. [2] and Jurado-Oller et al. [3].

These two publications studied H_2_ production in Chlamydomonas mixotrophic cultures incubated under low light during several days; 25 days in the case of Scoma et al. [2] and 10 days in Jurado-Oller et al. [3]. The former report used an illumination of 20 µmol photons m^−2^ s^−1^, while the later used a range of light intensities (from 12 to 50 µmol photons m^−2^ s^−1^). For comparative reasons, we will refer here to the data from Jurado-Oller et al. [3] obtained under 12 µmol photons m^−2^ s^−1^ since this is the condition that they examined the most. Note that, some other important differences between both publications' experimental set-ups could explain some discrepancies. Scoma et al. [2] used a D1 mutant, twice more concentration of acetate than the standard formulation of the TAP media, purged cultures, an initial cell concentration of 40–80 mg chl. L^−1^, and a ratio gas/liquid of 1.4 in the bioreactors. While Jurado-Oller et al. [3] used a strain without any photosynthetic phenotype (704), a standard TAP media recipe, no purged cultures, an initial cell concentration of 10 mg chl. L^−1^, and a gas/liquid ratio of 0.4 in the bioreactors. All these factors can greatly influence H_2_ production patterns.

Scoma et al. [2] reported that sealed cultures were able to produce some H_2_ after 4 h (≈9–11 ml l^−1^), and afterwards no more H_2_ production was observed during the next 10 days (this 10 days period is termed as Phase 1 by these authors). AQThese data are in good agreement with those published by Jurado-Oller et al. [3], who reported H_2_ production in sealed cultures after 24 h (≈7 ml l^−1^). Both studies reported that H_2_ production lasted less than 24 h, and afterwards no more H_2_ production was observed during the next 10 days. In the report of Jurado-Oller et al. [3], the headspaces of the bioreactors were not purged and atmospheric oxygen concentrations were present at the beginning of the experiment indicating that in the presence of acetate, low light-incubated cultures can quickly reach anoxia and produce H_2_ very fast. The earlier time at which H_2_ production was observed in Scoma et al. [2], relative to Jurado-Oller et al. [3] (4 vs 24 h), could be reflecting the fact that the earlier report used purged cultures and high cell concentration cultures, which allow a faster establishment of anaerobiosis.

Very interestingly, Scoma et al. [2] observed a second H_2_ production phase (Phase 2) not observed by Jurado-Oller et al. [3]. This H_2_ production phase occurred after 250 h of the establishment of the hypoxic conditions (around 10 days), and was sustained for about 14 days. Authors describe the H_2_ production observed during Phase 1 as “traces of H_2_” and production during Phase 2 as a “sharp H_2_ accumulation.” However, these differences in the H_2_ production rates should be considered more carefully. According to some of the published data ([2]; Figure 4), the H_2_ accumulation level about 125 h after the beginning of Phase 2 is lower than the initial H_2_ accumulation observed during the first 25 h of Phase 1. Unfortunately, the experiments performed by Jurado-Oller et al. [3] were stopped after 10 days, which is the precise time where Scoma et al. [2] observed their Phase 2 of H_2_ production, making impossible any comparison between these two reports in this regard.

Importantly, while Jurado-Oller et al. [3] concluded that H_2_ production is mostly linked to the PSII-independent pathway, Scoma et al. [2] concluded that H_2_ production during Phase 2 is primordially due to the PSII-dependent pathway. However, the report published by Scoma et al. [2] does not provide any conclusive data supporting this statement. Indirectly, Scoma et al. [2] based this affirmation on three facts: (1) starch accumulation is prevented in the cultures; (2) H_2_/CO_2_ ratios were lower in the presence of DCMU; (3) Fv/Fm values decreased during H_2_ production. However, under our understanding, none of these facts demonstrates that H_2_ production in these cultures is linked to the PSII activity. Moreover, a decrease of Fv/Fm during H_2_ production is not incompatible with a PSII-independent production. Interestingly, they reported almost no inhibition of the H_2_ production when adding DCMU ([2]; Figure 4b and Table 2), which is indicative of a dominant PSII-independent H_2_ production. Data represented in Figure 4b [2] illustrate how H_2_ is being produced in the presence of DCMU at the same time and to the same extent as the control cultures. Strikingly, authors increased light intensity in cultures containing DCMU (but not in control cultures) at the same time as Phase 2 started, which makes the interpretation of the data difficult.

On the other hand, Jurado-Oller et al. [3] stated that H_2_ production is mostly PSII-independent in aerated cultures since cultures treated with DCMU were able to produce up to 81% of H_2_ relative to control cultures (aeration was performed every 24 h by deliberately opening the bioreactors in a sterile atmosphere). Note that in the report of Jurado-Oller et al. [3], control cultures incubated in dark showed a substantially lower H_2_ production than cultures incubated in low light, indicating a minor role for the fermentative H_2_ production pathway operating during low light conditions. As explained in more detail below, the contribution of the PSII-independent pathway (deduced from the DCMU treatments) could be underestimated when oxygen availability of the cultures is very limited. Different operating conditions can enhance (or limit) PSII-independent vs -dependent H_2_ production, but undoubtedly, the H_2_ production observed in aerated cultures by Jurado-Oller et al. [3] is mainly linked to a PSII-independent pathway.

In Scoma et al. [2] and in the commentary article of Scoma and Toth (2017, Biotechnology for Biofuels), authors stated that under their experimental conditions, acclimation to hypoxia of the cultures required up to 10 days.

This concept of “acclimation” could be subjective and not easy to understand, since authors were able to detect H_2_ production after 4 h, implying that hypoxia was already established. Moreover, one might expect that cells incubated for up to 25 days (600 h) under hypoxic conditions could have their viability very compromised. In this regard, it is interesting to note that according to Figure 2 of Scoma et al. [2], oxygen is accumulated over 25 days in the headspace of the cultures incubated under 20 PAR (initial cell concentration of 40 mg chl. L^−1^), which could explain why cells were able to survive for such a long period. On the other side, Jurado-Oller et al. [3] reported that cultures containing four times less cells and incubated under 12 and 22 PAR showed no oxygen accumulation over 10 days (Figure 1b, in Jurado-Oller et al. [3]); moreover, the cell viability of these cultures was very compromised after this period (data not shown).

Finally, both publications reported that the starch reserves are not mobilized during H_2_ production, unlike in S-depleted cultures. On the contrary, the starch reserves increased during H_2_ production indicating that H_2_ production under these conditions is not linked to the mobilization of the starch reserves. Scoma et al. [2] reported a decrease in the protein content concomitant with the H_2_ production, which could potentially support PSII-independent production. On the other hand, Jurado-Oller et al. [3] suggested that acetate assimilation/dissimilation may be linked (directly or indirectly) to the PSII-independent H_2_ production during these conditions. This suggestion is based on the correlation of acetate uptake and H_2_ production when using DCMU (see below for a more detailed explanation). Although H_2_ production rates are not directly proportional to the acetate uptake rates, the former is impaired when the latter is also severely impaired. A tentative metabolic model is proposed by Jurado-Oller et al. [3], trying to describe how acetate assimilation/dissimilation can contribute to H_2_ production, linking acetate uptake with the TCA and glyoxylate cycles and with a non-photochemical reduction of the photosynthetic electron chain. A similar model was previously proposed by others [4]. However, these models are lacking any experimental evidence so far.

### The contribution of the PSII-dependent and -independent pathways to H_2_ production in the presence of acetate

The PSII inhibitor DCMU is broadly used to determine the respective contribution of the two H_2_ photoproduction pathways present in Chlamydomonas. If DCMU is added to cultures under conditions promoting H_2_ production, an inhibition of the H_2_ production is observed. This rate of inhibition is used to determine the contribution of the PSII-dependent and -independent pathways. Through this approach, several publications have evaluated the contribution of the PSII-dependent pathway in mixotrophic nutrient-depleted [5–9] and -replete cultures [2, 3, 10, 11]. Interestingly, very different values have been assigned, in both nutrient-replete and -depleted medium, to the contribution of the PSII-independent pathway, ranging from 0 to 100% of the total H_2_ production (Table 1). The contribution of the PSII-independent pathway has been largely studied in S-depleted cultures, and it is generally assumed that this pathway contributes to about 10–20% of the total H_2_ production in this medium [7, 9, 12]. However, it has been reported that several parameters greatly affect the PSII-independent contribution in S-depleted cultures when using DCMU [7, 8, 13]. Two parameters seem to be crucial for the differences in H_2_ production in these cultures: (1) the time at which DCMU is added to the cultures (right after S depletion or few days after S depletion) [7, 8, 13]; and (2) the cell density of the cultures [8]. The major contribution of the PSII-independent pathway is obtained when low cell density cultures are used or when DCMU is added few days after S depletion.

**Table 1:** Comparison of the in vivo PSII-independent contribution to H_2_ production under different conditions

Reference	In vivo PSII-independent contribution^a^ (%)	Media	Strain	Cell density^b^	Purged cultures	PAR^c^	Notes
Healey [11]	100	NR	*C. moewusii* ICC 97			300 (lux)	Dark–light cycle adaptation
	100	NR	*C. dysosmos* ICC 242			400 (lux)	Dark–light cycle adaptation
Gibbs et al. [10]	18	NR	F60		Yes	100 W/m^2^	Dark adaptation (2 h) DCMU and acetate added simultaneously Acetate uptake is inhibited by 91% in the presence of DCMU
Scoma et al. [2]	86^d^	NR	L159I-N230Y (D1 mutant)	80	Yes	100	DCMU added after 150 h 2X acetate in TAP formulation In cultures without DCMU, O_2_ accumulated in the headspaces
Jurado-Oller et al. [3]	21	NR	704	10	No	12	DCMU added at 0 h Atmospheric O_2_ level when DCMU is added O_2_ levels remained very low after 24 h
	36	NR	704	10	Yes	12	DCMU added at 0 h
	81	NR	704	10	No	12	DCMU added at 0 h Atmospheric O_2_ level when DCMU is added Cultures aerated every 24 h
Hemschemeier et al. [8]	0	-S	cc124	20	No	100	DCMU immediately after S depletion
	40	-S	cc124	20	No	100	DCMU added 17 h after S depletion
	80–65	-S	cc124	20	No	100	DCMU added during H_2_ production phase H_2_ measured by MIMS
	40	-S	cc124	27	No	100	DCMU added during H_2_ production phase H_2_ measured by MIMS
	100	-S	cc124	17	No	100	DCMU added during H_2_ production phase H_2_ measured by MIMS
Fouchard et al. [7]	≈0	-S	cc124	5 × 10^6^ cells/ml	Yes	110	DCMU immediately after S depletion O_2_ levels near 0% when DCMU is added
	20	-S	cc124	5 × 10^6^ cells/ml	Yes	110	DCMU was added 24 h after S depletion O_2_ levels near 10% when DCMU is added
Laurinavichene et al. [13]	51	-S	cc124	20–28	Yes	30	DCMU added after 46 h of S depletion H_2_ production is maximal as this time
	32	-S	cc124	20–28	Yes	30	DCMU added after 70 h of S depletion
	28	-S	cc124	20–28	Yes	30	DCMU added after 94 h of S depletion
Chochois et al. [12]	10	-S	330	4 × 10^6^ cells/ml	Yes	200	DCMU added 24 h after S depletion
Antalet al. [15]	30	-S	cc124	4 × 10^6^ cells/ml	Yes	25	
Philips et al. [5]	100	-N	cc124	5 × 10^6^ cells/ml	No	60	DCMU added 72 h after N depletion
Volgusheva et al. [6]	26	-Mg	cc124	7	No	80	DCMU added 8 days after Mg depletion O_2_ levels near 0 when DCMU is added

*NR* Nutrient Replete

^a^Measured as the % of H_2_ production in the presence of DCMU (relative to control cultures)

^b^In mg chl. L^−1^ unless otherwise indicated

^c^Photosynthetic active radiation (PAR) in µmol photons m^−2^ s^−1^, unless otherwise indicated

^d^Data according to Table 2 in original publication

Similarly, Jurado-Oller et al. [3] also reported that in mixotrophic nutrient-replete cultures incubated in low light, the addition of DCMU caused very different effects on H_2_ production depending on the growth conditions. In this case, oxygen availability of the cultures (provided by aeration) greatly increased the contribution of the PSII-independent pathway on H_2_ production (81%) when compared to non-aerated cultures (21%) or purged cultures (36%). Recently, in a commentary article of Scoma and Toth (Biotechnology for Biofuel 2017), these data were considered as contradictory, but under our point of view, they are reflecting how cultures under different physiological conditions respond differently to a PSII inhibitor, similarly to what has been reported in S-depleted media. From these data, Jurado-Oller et al. [3] proposed that the effect of DCMU on H_2_ production in mixotrophic cultures incubated under low light is modulated by the presence of oxygen in the cultures; the more the oxygen the less the inhibition. In contrast to the inhibition caused by DCMU observed by Jurado-Oller et al. [3] in non-aerated cultures (79% inhibition of the total H_2_ production), Scoma et al. [2] observed very little inhibition of H_2_ production when using DCMU in sealed cultures (14% inhibition of the total H_2_ production according to the data presented in Table 2, [2]). Interestingly, Jurado-Oller et al. [3] described no oxygen accumulation in the headspaces of non-aerated cultures incubated under 12 PAR (Figure 1b in Jurado-Oller et al. [3]); however, Scoma et al. [2] reported the presence of oxygen in the headspaces of the cultures (Figure 2, [2]).

Since PSII activity is dispensable to obtain 81% of total H_2_ production in aerated cultures incubated under low light, Jurado-Oller et al. [3] proposed that the PSII-independent pathway is the primordial via to produce H_2_ in these cultures. Moreover, Jurado-Oller et al. [3] also demonstrated that acetate uptake is greatly dependent on oxygen availability. Hence, the effect of DCMU can also affect greatly the acetate uptake rates depending on the different oxygenation regimes of the cultures. Cultures supplemented with DCMU under aeration regime were essentially unaffected in their capacity to uptake acetate (relative to control cultures), whereas non-aerated and purged cultures presented a severe impairment of the acetate uptake. Based on these facts (acetate uptake and H_2_ production in the presence of DCMU), Jurado-Oller et al. [3] suggested that the H_2_ production in nutrient-replete cultures under low light could be linked (directly or indirectly) to the acetate uptake rates. Note that, as deduced from purged cultures, the releasing of the H_2_ partial pressure (without providing oxygen) in the presence of DCMU is not greatly contributing in increasing neither the contribution of the PSII-independent H_2_ production (36%) nor the acetate uptake rates (acetate uptake is essentially blocked) (Figure 3 in Jurado-Oller et al. [3]). This is indicating that oxygen availability, and not the releasing of the H_2_ partial pressure, is affecting these two processes. Overall, the data provided by Jurado-Oller et al. [3] revealed that the inhibitory effect caused by DCMU on H_2_ production in mixotrophic cultures incubated under low light could not be entirely linked to the lack of electrons provided by the PSII activity, but mainly to an indirect effect related to oxygen availability.

The data provided by Jurado-Oller et al. [3] and their interpretation could be in partial agreement with the data reported for S-depleted cultures. The different effects of DCMU on H_2_ production in S-depleted cultures were explained based on the capacity of the cultures to accumulate starch before the addition of DCMU, since this inhibitor blocks also starch accumulation [7]. Starch is accumulated during the oxygenic phase and constitutes the main source of reductants feeding the PSII-independent H_2_ production once the cultures reach the anoxic phase. Authors proposed that starch accumulation before the H_2_ production phase is not impaired if the addition of DCMU takes place few days after S depletion or if low cell density cultures are used [7, 8]. Under these conditions, the contribution of the PSII-independent pathway is more significant. Cell culture density could influence effective light intensity and thereby PSII activity. Thus, authors proposed that the PSII activity is essential to accumulate starch during the aerobic phase, which in turn will contribute to the PSII-independent H_2_ production. However, an alternative possibility cannot be ruled out, which is not based on the PSII activity per se but on the oxygen availability and acetate uptake rates. Oxygen availability could allow acetate uptake, which in turn will also impact starch accumulation.

Note that those conditions favoring PSII-independent production in S-depleted cultures are precisely conditions where oxygen availability could be higher. On the contrary, severe anoxic conditions can lead to very low acetate uptake, low starch levels, and very low PSII-independent H_2_ production. In general, there is a good correlation in the literature between the oxygen availability of the cultures and the degree of contribution of the PSII-independent pathway (Table 1) in both nutrient-replete and -deplete cultures. Conditions favoring anoxia such as purged cultures, high cell concentration, early addition of DCMU in S-depleted cultures, or different mutations causing low O_2_ evolution can result in an underestimation of the PSII-independent pathway.

## Conclusions

Incubation of mixotrophic nutrient-replete cultures under restricted light conditions lead to a rapid (<24 h) accumulation of H_2_ [2, 3]. Interestingly, these cultures can show a second H_2_ production phase after prolonged incubation (>10 days) under hypoxic conditions [2]. This strategy could be an alternative way to produce H_2_ to those based on nutrient-depleted conditions with the additional advantage of producing biomass simultaneously [2, 3]. Unfortunately, compared with the production in S-depleted cultures, H_2_ production under this condition is lower, which makes even more difficult any potential biotechnological application. Still, the number of publications studying H_2_ production under mixotrophic nutrient-replete cultures is very scarce and more knowledge could be gained in the future. In addition, in mixotrophic low light cultures, the release of the H_2_ pressure and/or the supplementation with additional acetate can lead to a substantial optimization of the H_2_ production [3].

It has been proposed that in mixotrophic S-depleted cultures, the activity of the PSII during the aerobic phase is indispensable for H_2_ production [7, 8]. Yet, as demonstrated by Jurado-Oller et al. [3], the PSII activity is dispensable for H_2_ production in mixotrophic nutrient-replete cultures incubated under low light and under aeration. However, under nutrient-replete conditions, the relative contributions of the PSII-dependent and -independent pathways are still a matter of debate since their contribution may vary with the culture conditions. Further studies will be necessary to demonstrate conclusively the relative contribution of these two pathways in this condition. In any case, the starch reserves do not seem to be linked to H_2_ production [2, 3, 14]. Catabolism of proteins [2, 14] or acetate assimilation/dissimilation [3, 4, 10] are potential processes that can provide reductive power for PSII-independent H_2_ production. However, conclusive results to characterizing the main electron source used for PSII-independent H_2_ production under this condition are still missing.

In the available literature, the use of DCMU to calculate the contribution of the two photosynthetic H_2_ production pathways can generate a large variety of effects, depending on the specific growth conditions. It is possible that oxygen levels in the cultures can determine the degree of inhibition caused by DCMU [3]. The effect caused by DCMU could not only be linked to the inhibition of the PSII activity per se and to the concomitant loss of electrons that can enter in the photosynthetic chain, but also to the elimination of the main source of oxygen in sealed cultures. Oxygen is indispensable for the uptake of acetate [3], which in turn can also impair starch accumulation. Moreover, the lack of oxygen can also have an important impact under illumination conditions on chlororespiration, photorespiration, and the Mehler reaction. All these factors could be linked to (or influence) H_2_ production in Chlamydomonas under different conditions, which make difficult the interpretation of the results regarding the PSII-independent contribution to H_2_ production when using DCMU. In any case, the PSII-independent contribution could be greatly underestimated when adding DCMU to cultures where oxygen is severely depleted or when starch accumulation is prevented in the case of S-depleted cultures.
